# Correction: Synergistic, multi-level understanding of psychedelics: three systematic reviews and meta-analyses of their pharmacology, neuroimaging and phenomenology

**DOI:** 10.1038/s41398-025-03731-7

**Published:** 2025-11-13

**Authors:** Kenneth Shinozuka, Katarina Jerotic, Pedro Mediano, Alex T. Zhao, Katrin H. Preller, Robin Carhart-Harris, Morten L. Kringelbach

**Affiliations:** 1 Centre for Eudaimonia and Human Flourishing, Linacre College, University of Oxford, Oxford, UK; 2 Department of Psychiatry, University of Oxford, Oxford, UK; 3 Oxford Mathematics of Consciousness and Applications Network (OMCAN), University of Oxford, Oxford, UK; 4 Department of Computing, Imperial College London, London, UK; 5 Department of Statistics and Data Science (Alumnus), The Wharton School, University of Pennsylvania, Philadelphia, PA USA; 6 Departments of Psychiatry, Neuroscience, and Psychology, Yale University, New Haven, CT USA; 7 Department of Psychiatry, Psychotherapy and Psychosomatics, Psychiatric University Hospital Zurich, University of Zurich, Zurich, Switzerland; 8 Centre for Psychedelic Research, Imperial College London, London, UK; 9 Department of Neurology, University of California, San Francisco, CA USA; 10 Department of Neurology, Psychiatry and Behavioral Sciences, University of California, San Francisco, CA USA; 11 Department of Clinical Medicine, Center for Music in the Brain, Aarhus University, Aarhus, Denmark

**Keywords:** Neuroscience, Human behaviour, Pharmacology

Correction to: *Translational Psychiatry* 10.1038/s41398-024-03187-1, published online 04 December 2024

The meta-analysis of the 11-dimensional Altered States of Consciousness (11D-ASC) included three studies that contained duplicated data from other papers: Duerler et al. (2021), Lewis et al. (2017), and Preller et al. (2017). When these studies are removed from the analysis, the results are generally similar to the previously reported findings. Out of 33 comparisons (3 doses x 11 dimensions) between LSD and psilocybin, one becomes insignificant (insightfulness, medium dose), while one becomes more significant (disembodiment, medium dose). Note that the 5D-ASC meta-analysis did not include any duplicates, hence the results of that meta-analysis are unaffected.

We also wish to acknowledge two previous meta-analyses on the phenomenology of LSD and psilocybin, respectively, by Hirschfeld and colleagues:

1. Hirschfeld, T., Prugger, J., Majic, T., Schmidt, T.T. Dose-response relationships of LSD-induced subjective experiences in humans (2023). Neuropsychopharmacology, 48, 1602-1611.

2. Hirschfeld, T., Schmidt, T.T. Dose-response relationships of psilocybin-induced subjective experiences in humans (2021). Journal of Psychopharmacology. 35(4), 384-397.

The original article has been corrected.

